# High-signal bladder urine at *T*_1_-weighted MR imaging performed 1–7 days after a prior gadolinium-enhanced MRI: prevalence and correlation with renal function

**DOI:** 10.1259/bjro.20180030

**Published:** 2019-05-24

**Authors:** Paul Foran, Sinchun Hwang, Yousef Mazaheri, David M. Panicek

**Affiliations:** 1 Departments of Radiology, Memorial Sloan Kettering Cancer Center, New York, USA; 2 Weill Medical College of Cornell University, New York, NY, USA; 3 Department Medical Physics, Memorial Sloan Kettering Cancer Center, New York, USA

## Abstract

**Objective:**

To determine the prevalence of high-signal bladder urine at *T*_1_ weighted MRI performed 1–7 days after injection of gadolinium-based contrast material and to assess for correlation with altered renal function.

**Methods:**

The study group consisted of 267 patients who underwent MRI that included the bladder 1–7 days after a prior gadolinium-enhanced MRI. A control group consisted of 200 patients who underwent pelvic MRI and had not received gadolinium-based contrast material within the prior month. One reader recorded the relative *T*_1_ weighted signal intensity of bladder urine and calculated the estimated glomerular filtration rate (eGFR) for each patient. A positive scan was defined as one with bladder urine *T*_1_ weighted signal higher than that of muscle.

**Results:**

25 (9%) of 267 study group scans were positive; this included 68% ( *n* = 19) of scans obtained 12–24 h after gadolinium-based contrast material administration, 21% ( *n* = 3) after 25–36 h, 7% ( *n* = 2) after 37–48 h, and 3% ( *n* = 1) after 49–72 h. No positive scan occurred after 72 h or in the control group. Mean eGFR in positive scans obtained more than 36 h after gadolinium-based contrast material administration was significantly lower than in negative scans in the same timeframe (37 *vs* 76 ml/min, respectively; *p* = 0.01).

**Conclusion:**

High *T*_1_ weighted signal in bladder urine occasionally is present on MRI performed up to 3 days after gadolinium-based contrast material administration, presumably reflecting residual excreted gadolinium-based contrast material. When visible more than 36 hours after gadolinium-based contrast material administration, such increased signal is associated with low eGFR.

**Advances in knowledge:**

Increased signal is occasionally present in bladder urine at MRI performed up to 3 days after gadolinium-based contrast material administration. When higher signal is visible more than 36 hours after contrast administration, it is associated with decreased eGFR.

## Introduction

Gadolinium-based contrast material (GBCM) is commonly administered during MRI examinations. GBCM is predominantly excreted by the kidneys, with a circulating half-life of approximately 1.5 h in patients with normal renal function.^1–5^ These compounds are eliminated through the kidneys; as a result, in patients with renal disease, the rate of elimination is reduced.^6,7^ Studies have shown a substantial increase in the mean elimination half-life of gadolinium as the degree of renal insufficiency increases; in one study, *e.g.* patients with severe renal insufficiency showed a more than 30-fold increase in the half-life elimination of GBCM.^8^

Several studies have demonstrated the accumulation of gadolinium within brain^9^ and bone.^10^ Animal studies have also demonstrated a correlation between signal intensity(SI) ratio increase and gadolinium deposition in neural tissue.^11,12^ At MRI, bladder urine normally demonstrates the signal characteristics of simple fluid, including low SI compared to that of muscle on *T*_1_ weighted (*T*_1_W) images. We occasionally observed increased *T*_1_W-SI in bladder urine on MR images obtained within a few days after a previous GBCM-enhanced MRI. As GBCM is expected to be >95% excreted within 24 h of administration in patients with normal renal function,^13^ we postulated that high *T*_1_W-SI in bladder urine visible after this time may be caused by delayed excretion of GBCM secondary to impaired renal function. Therefore, we performed this study to determine the prevalence of increased *T*_1_W-SI in bladder urine within a week after GBCM administration and to assess for a correlation with altered renal function.

## Methods and materials

### Patient population

**Figure 1.**
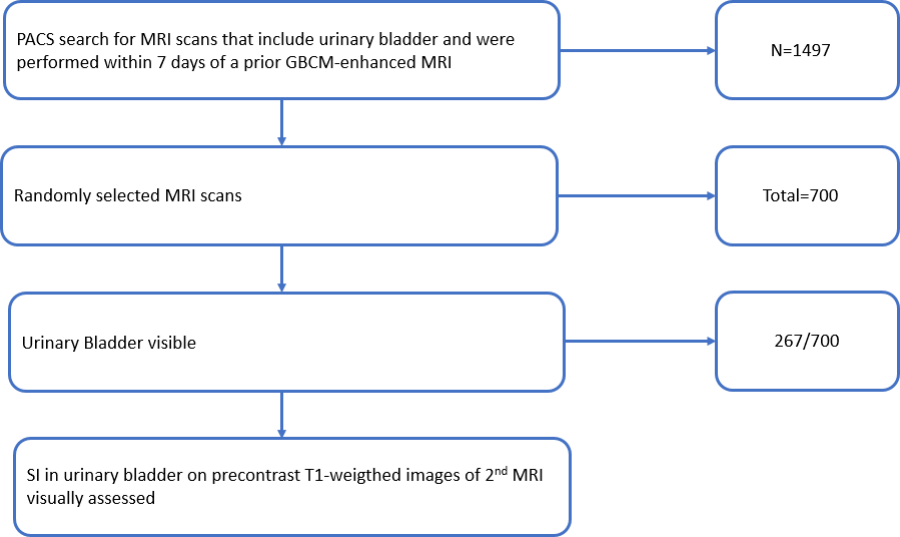
Flowchart of study.

This retrospective study was conducted at our tertiary cancer center after approval by the institutional review board, which waived the need for informed consent. The selection of patients and analysis of scans in the study group are outlined in Figure 1. A computerized search of the picture archiving and communication system (PACS) was conducted for patients who, between January 2007 and December 2013, underwent MRI that potentially included the urinary bladder 1–7 days after a prior GBCM-enhanced MRI. Such scans included MRI of spine, pelvis, hip and femur. Patients under the age of 18 years were excluded because their height at the time of the MRI is often unavailable in the medical records, precluding accurate calculation of estimated glomerular filtration rate (eGFR). 100 MRI scans from distinct patients were randomly selected for each of seven groups; the groups were based on the number of days (1– to 7) between the initial GBCM administration and the second MRI. For each study patient, the number of hours between the initial GBCM administration and the second MRI was calculated. A control group consisted of pelvic MRI examinations performed in 200 patients who received no GBCM within 30 days prior to the MRI.

### Image analysis

The study and control group images were reviewed in random order. One of two radiologists (one faculty radiologist with 9 years experience, and one oncologic imaging fellow) assessed whether the urinary bladder was visible on pre-contrast *T*_1_W images. If visible, the largest elliptical region of interest (ROI) that would fit entirely within the visible bladder lumen (the entire bladder was often not always within the field of view) was placed, taking care to exclude the bladder wall and any intravesical air. For this ROI, the SI of urine relative to muscle and of urine relative to fat on those images was recorded as well as technical parameters of the sequence: field strength, repetition time (TR) and echo time (TE). Fast relaxation fast spin-echo (FRFSE) *T*_1_W sequences were excluded due to the high SI of fluid that can be produced with that sequence.^14^

**Figure 2.**
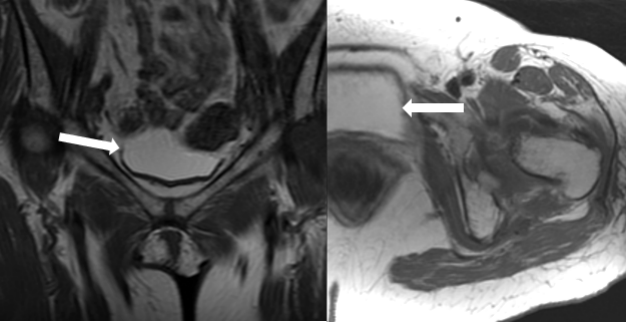
71-year-old female with metastatic pulmonary adenocarcinoma whopresented with lower back pain and underwent gadolinium-enhanced MRI of spine(not shown). Two days later, MRI of pelvis was performed. (a) Coronal and (b)axial precontrast T1W images demonstrate high T1W-SI in bladder urine (whitearrows), equal in SI to that of nearby fat.

A scan was deemed positive if bladder urine had *T*_1_W-SI higher than that of muscle at visual inspection, and negative if the *T*_1_W-SI was equal to or less than that of muscle (Figure 2). High *T*_1_W-SI in the bladder due only to ghosting or other artifact was not considered a positive scan. The readers were blinded to the number of days since prior GBCM injection.

The dose and type of GBCM administered at the initial MRI subsequently were recorded.

### Estimation of renal function

The estimated glomerular filtration rate (eGFR) for each patient in the study and control groups was calculated using the Chronic Kidney Disease Epidemiology Collaboration (CKD-EPI) equation^15^. Compared to the Modification of Diet in Renal Disease (MDRD) equation, this equation has been shown to be more accurate for eGFR levels between 60–120 ml/min, and as accurate for eGFR levels below 60 ml/min.^16^ For each patient, the serum creatinine level obtained closest to the time of GBCM injection, or in control scans to the time of the MRI scan, was obtained from the electronic medical record.

### Statistical analysis

The differences in eGFR of positive and negative scans occurring within various timeframes after GBCM administration (12–24 h, 25–36 h, 37–48 h, 49–72 h; and 12–36 h and 37–72 h) were compared using the Mann–Whitney *U* test.

## Results

### Study scans

The initial search yielded 1497 scans. Among the randomly selected 700 MRI scans, the urinary bladder was visible in 267 scans [128 male, 139 female; mean age, 59 years (range, 18–91 years)]; these scans constitute the study group. The administered GBCM was gadopentetate dimeglumine (Magnevist; Bayer Healthcare, Wayne, NJ) in 263 scans; the mean dose was 14.9 ml (range, 6–20 ml). 10 ml gadoxetate disodium (Eovist; Bayer Healthcare, Wayne, NJ) was administered in each of 3 scans, and 18 ml gadobenate dimeglumine (MultiHance; Bracco Diagnostics, Princeton, NJ) in 1 scan.

Regarding the second MRI performed, 214 (80%) were of the spine (80%), 50 (19%) of hip with internal derangement protocol, 2 (0.8%) of pelvis and 1 (0.4%) of lumbar plexus. The MRI scans were performed at 1.5 T in 251 scans (94%), with a mean TR of 478 ms (range, 317–750 ms) and a mean TE of 9 ms (range, 3–20 msec); and at 3 T in 16 scans (6%), with a mean TR of 631 ms (range, 400–850 ms) and a mean TE of 10 ms (range, 6–20 ms).

A serum creatinine level was available in all 267 study patients. The median time interval between serum creatinine level and initial MRI was 2 days (range, 0–1744 days).

**Figure 3.**
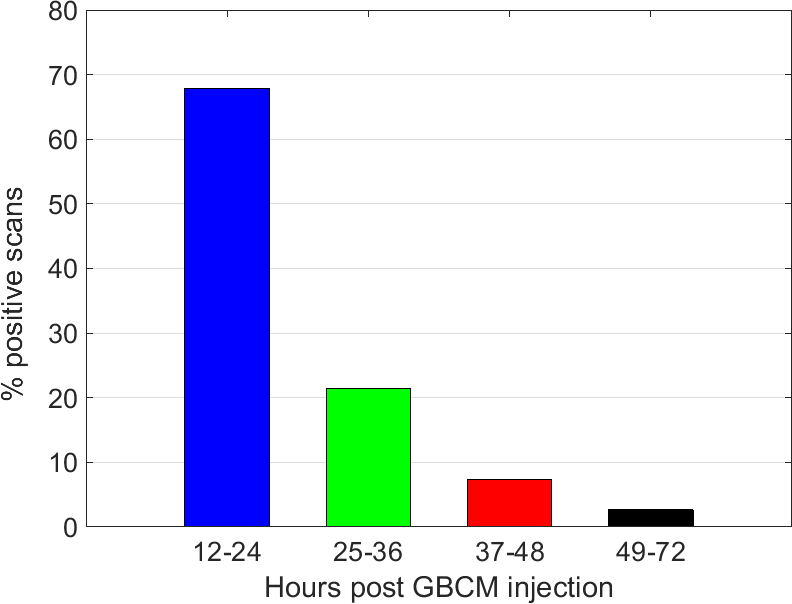
Number of patients with high bladder urine T1W-SI(positive scans) as a function of time interval after GBCM administration.

In the study group, scans were scored as positive in 25 (9%) of 267 patients. The percentage of positive scans decreased with increasing time from prior GBCM injection (Table 1; Figure 3). For analysis, the study patients were grouped according to the number of hours between prior GBCM injection and the second MRI. No patient was imaged sooner than 12 h after prior GBCM injection, and no positive scan was encountered if more than 72 h had elapsed from previous GBCM injection. All positive scans occurred after gadopentetate dimeglumine (Magnevist) administration. Among the 25 positive scans, the median number of days between the serum creatinine level and initial MRI was 0 days (range, 0–55 days).

**Table 1.** Mean eGFR for patients with and without high bladder urine *T*_1_W SI, for each time period after GBCM injection.

**Study scans**								
**Time after GBCM injection (hours**)	**# of patients**	**# of positive scans (%**)	**Mean eGFR (ml/min) for positive scans (range**)		**Mean eGFR (ml/min) for negative scans (range**)		*p*-value	*p*-value
**12–** **24**	28	19 (68)	80 (33–128)	81 (33–128)	96 (63–135)	90 (31–135)	0.25	0.34
**25–** **36**	14	3 (21)	89 (38–126)		85 (31–133)		0.77	
**37–** **48**	27	2 (7)	43 (25–61)	37 (23–61)	81 (28–120)	76 (28–131)	0.06	0.01
**49–** **72**	38	1 (3)	23		74 (36–131)		0.10	
**73–** **96**	35	0 (0)	-	-	77 (25–138)	73 (25–138)	-	-
**97–** **120**	37	0 (0)	-		76 (37–130)		-	
**121–** **144**	36	0 (0)	-		69 (34–115)		-	
**145–** **168**	23	0 (0)	-		79 (30–116)		-	
**169–** **192**	29	0 (0)	-		65 (31–133)		-	

GBCM, gadolinium-based contrast material; SI, signal intensity; eGFR, estimated glomerular filtration rate.

**Figure 4.**
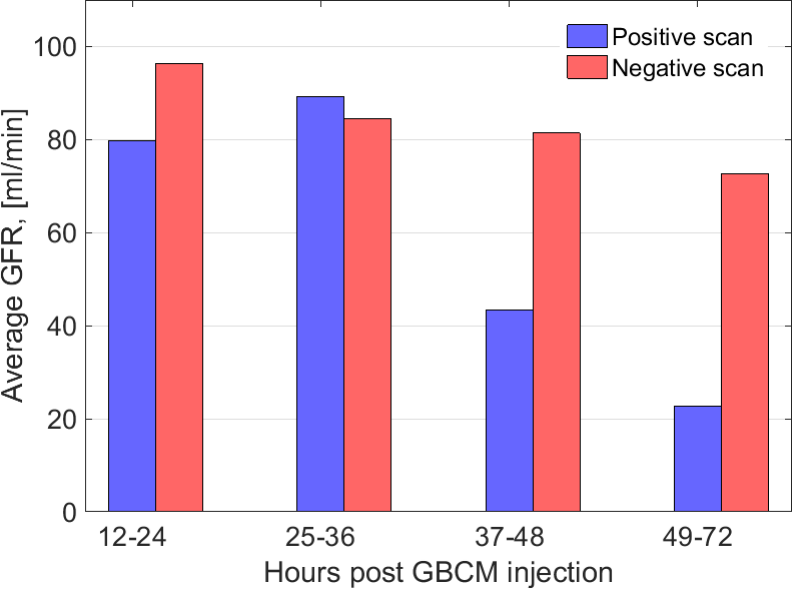
Mean eGFR in patients with high bladder urine T1W-SI(positive scans) and patients without high bladder urine T1W-SI (negativescans) as a function of time interval after GBCM injection. The mean eGFR wasmarkedly lower in scans obtained more than 36 hours after GBCM injection(p=0.01).

The mean eGFR were similar in positive and negative scans imaged less than 36 h after GBCM injection (87 ml/min and 90 ml/min, respectively; *p* = 0.34) (Figure 4). However, the mean eGFR of the three patients who had high *T*_1_W-SI between 37 and 72 h was markedly lower than those who did not have high *T*_1_W-SI in the same timeframe (37 *vs* 76 ml/min, respectively; *p* = 0.01). The etiology of renal impaired function in the study group was difficult to analyze due to the patients’ complex oncologic treatment regimens including multiple lines of chemotherapy, radiation therapy, and complications from medical and surgical interventions.

### Control scans

The urinary bladder was visualized in each of the 200 control MRI scans, which were obtained in 100 males and 100 females [mean age, 60 years (range, 18–88 years)]. 100 scans (50%) were of the prostate, 42 of musculoskeletal pelvis with tumor protocol (21%), 33 of bladder (17%), 24 of gynecologic pelvis (12%), and 1 of hip with internal derangement protocol (0.5%). MRI was performed at 1.5 T in 130 scans (65%), with a mean TR of 553 ms (range, 367–817 ms) and a mean TE of 9 ms (range, 5–25 ms); and 70 scans (35%) were performed at 3 T with a mean TR of 675 ms (range, 483–906 ms) and a mean TE of 8 ms (range, 6–15 ms). No control scan showed bladder urine with *T*_1_W-SI higher than that of muscle.

A serum creatinine level was available in 168 (84%) of 200 patients in the control group. The median time interval between serum creatinine level and initial MRI was 2 days (range, 0– to 2587 days). The mean eGFR was 83 ml/min (range, 28–132 ml/min).

## Discussion

In this study, high SI was visible in bladder urine on *T*_1_W images on numerous patients in the first 72 h after GBCM injection, presumably reflecting the presence of excreted GBCM in the urine. Even up to 36 h after GBCM injection, this finding was not associated with alterations in renal function. However, a small number of patients with high SI in urine more than 36 h after GBCM injection had significantly lower mean eGFR than patients imaged in the same timeframe who did not demonstrate this finding, suggesting an association with impaired renal function in our oncologic population.

Other potential causes of high *T*_1_W-SI of bladder urine, unrelated to GBCM, should be considered. These include hematuria and macromolecules in the urine secondary to infection, neoplasm, bladder outlet obstruction, neurogenic bladder, diet, medications and intervention. Given the retrospective nature of this study, it was not possible to systematically assess for these entities in our patient population. In a study to assess the possible clinical significance of high *T*_1_W-SI in bladder urine, Rosenkrantz et al^17^ reported high bladder urine *T*_1_W-SI in 4 (7%) of 56 patients with prostate cancer and 6 (4%) of 160 male control subjects. None of these patients received GBCM within 6 months prior to the MRI at their institution although history of prior GBCM injection at other facilities would not have been available. Among the prostate cancer patients, there was no association between high bladder *T*_1_W-SI and abnormal urinalysis or impaired renal function. Based on these observations, the authors suggested that high *T*_1_W-SI of bladder urine at prostate MRI is most likely an incidental finding of doubtful clinical significance. However, in our study no high *T*_1_W-SI was observed in bladder urine in any of the control scans or in scans obtained more than 72 h after GBCM injection. This strongly suggests that high *T*_1_W-SI in bladder urine identified in our positive scans is related to residual excreted GBCM.

When apparent high *T*_1_W-SI is identified within the bladder, it is important to ensure that the sequence is truly *T*_1_W. For example, fast recovery fast spin echo (FRFSE) sequences have short TR yet produce high SI of fluid including normal bladder urine.^14^ The high SI produced in normal bladder urine on *T*_1_W FRFSE sequences could potentially be misinterpreted as abnormal. However, in such cases, awareness of the imaging characteristics of these sequences as well as observation of the high SI produced in fluid elsewhere in the image, such as cerebrospinal fluid in the spinal canal or synovial fluid in a hip joint, should alert the radiologist to the cause of the high SI in bladder urine.

After excluding technical and artifactual causes of high *T*_1_W-SI of bladder urine, the radiologist should attempt to ascertain a cause. A history of gross hematuria may be available. If GBCM has been administered in the 36 h preceding the current MRI, the findings of our study suggest that the abnormal SI can be attributed to excretion of GBCM in bladder urine. If GBCM has been recently administered but more than 36 h prior to the current MRI, this likely is due to delayed excretion of GBCM secondary to impaired renal function, and a new measurement of the patient’s serum creatinine level should be considered to evaluate the current renal function. This awareness is particularly important in caring for patients at increased risk for renal insufficiency, such as patients with diabetes or cancer. If there has been no recent GBCM administration and no gross hematuria is present, the study by Rosenkrantz et al suggests that high *T*_1_W-SI in bladder urine may be an idiopathic incidental finding of no clinical importance.^17^ The results of our study reinforce their suggestion.

This study has a few limitations, including the biases inherent in any retrospective study. Most important, chemical analysis of the bladder urine was not performed. Therefore, although we postulate that high *T*_1_W-SI of bladder urine was due to the presence of excreted GBCM, this was not proven. Other potential causes of high *T*_1_W-SI in bladder urine, such as hematuria and macromolecules in the urine secondary to infection, neoplasm, bladder outlet obstruction, neurogenic bladder, diet, medications and intervention, might have been responsible for this observation. Study scans were randomly selected for analysis; the results could have been different if a different patient population had been selected. In some studies, such as spine MRI, urinary bladder was partially included, potentially increasing the falsely negative cases. Only three scans were positive more than 36 h after GBCM injection, limiting the statistical power of the study for that subgroup. For a few patients, particularly those in the control group, the serum creatinine level used to calculate the eGFR was obtained months or years prior to the MRI, and thus may not have accurately represented the patient’s renal function at the time of MRI. However, this reflects current clinical practice. The ACR does not recommend assessing renal function in all patients. In our institution (Memorial Sloan Kettering Cancer Center), after careful screening of all outpatients by nursing staff, eGFR is evaluated only in those patients deemed at high risk for renal impairment. It was not possible to determine the time of last micturition for each patient before their MRI; therefore, high *T*_1_W-SI in bladder urine may have represented GBCM that was excreted many hours prior to the MRI. For example, this may have occurred in one of the positive scans, obtained 48 h after GBCM injection; the patient’s eGFR was 61 ml/min, and their bladder was markedly distended, suggesting urinary retention. The administered GBCM in this study (Magnevist, MultiHance and Eovist) are linear ionic GBCM. All cases of bladder *T*_1_-hyperintense signal occurred with Magnevist, a group I agent that has been associated with nephrogenic systemic fibrosis and is contraindicated in patients with eGFR <30 ml/min.^18^ Macrocyclic GBCM are increasingly used, particularly in patients with impaired renal function. However, given that Magnevist and macrocyclic agents are almost exclusively excreted by the kidneys, similar findings may be expected with their use. As per the 2018 ACR Manual on Contrast Media v. 10.3 ^18^ , patients at risk for nephrogenic systemic fibrosis (*i.e.* AKI or Stage 4/5 chronic kidney disease) can, at times, receive GBCA if the risks of administering a GBCA are less than the substantial risk of not performing a needed contrast-enhanced imaging procedure. In such cases, Group II GBCA are recommended. However, distinction between Group I, II and III GBCA was not made in the 2008 ACR Manual on Contrast Media and, considering that the cases in our study date back to 2007, some of the patients with eGFR <30 ml/min who received GBCA likely had a pressing clinical need for a contrast-enhanced MRI. Also, GBCA administration would have been consonant with the specific guidelines in effect at the time of imaging. At this temporal remove, it is not possible to elucidate the particular clinical scenario for each of these patients. Currently, high-risk patients may still receive GBCA in the appropriate clinical scenario but should receive Group II GBCA, agents which, similar to Magnevist, are almost exclusively excreted by the kidneys. Thus, similar findings of bladder *T*_1_ hyperintense signal are expected to be found in such patients.

In conclusion, high SI occasionally is visible in bladder urine on *T*_1_W MR images performed as long as 3 days after GBCM administration, presumably reflecting residual excreted GBCM. When visible more than 36 h after GBCM administration, such increased SI is associated with decreased eGFR. Therefore, when high *T*_1_W-SI of bladder urine is identified, it should not be assumed that this is secondary to blood or other protein material in the urine. Rather, one should first review the technical parameters to ensure that the sequence is truly a *T*_1_W sequence. Also, a history of recent gross hematuria should be sought. Then, one should determine whether the patient had received GBCM recently; if such had occurred more than 36 h earlier, a new serum creatinine level should be obtained for the patient unless they already are known to have substantial renal impairment.
